# Detection of Adulteration of Extra Virgin Olive Oil via Laser-Induced Breakdown Spectroscopy and Ultraviolet-Visible-Near-Infrared Absorption Spectroscopy: A Comparative Study

**DOI:** 10.3390/foods14020321

**Published:** 2025-01-18

**Authors:** Eleni Nanou, Marios Bekogianni, Theodoros Stamatoukos, Stelios Couris

**Affiliations:** 1 Department of Physics, University of Patras, 26504 Patras, Greece; e.nanou@iceht.forth.gr (E.N.); up1068704@ac.upatras.gr (M.B.); up1071016@ac.upatras.gr (T.S.); 2 Institute of Chemical Engineering Sciences (ICE-HT), Foundation for Research and Technology-Hellas (FORTH), 26504 Patras, Greece

**Keywords:** Laser-Induced Breakdown Spectroscopy—LIBS, UV-Vis-NIR absorption spectroscopy, extra virgin olive oil, edible oils, adulteration, machine learning

## Abstract

The fast detection of Extra Virgin Olive Oil (EVOO) adulteration with poorer quality and lower price vegetable oils is important for the protection of consumers and the market of olive oil from fraudulent activities, the latter exhibiting an increasing trend worldwide during the last few years. In this work, two optical spectroscopic techniques, namely, Laser-Induced Breakdown Spectroscopy (LIBS) and UV-Vis-NIR absorption spectroscopy, are employed and are assessed for EVOO adulteration detection, using the same set of olive oil samples. In total, 184 samples were studied, including 40 EVOOs and 144 binary mixtures with pomace, soybean, corn, and sunflower oils, at various concentrations (ranging from 10 to 90% *w*/*w*). The emission data from LIBS, related to the elemental composition of the samples, and the UV-Vis-NIR absorption spectra, related to the organic ingredients content, are analyzed, both separately and combined (i.e., fused), by Linear Discriminant Analysis (LDA), Support Vector Machines (SVMs), and Logistic Regression (LR). In all cases, very highly predictive accuracies were achieved, attaining, in some cases, 100%. The present results demonstrate the potential of both techniques for efficient and accurate olive oil authentication issues, with the LIBS technique being better suited as it can operate much faster.

## 1. Introduction

Olive oil, recognized as “liquid gold”, boasts several valuable nutritional constituents, such as high levels of mono-unsaturated fatty acids (oleic acid), vitamins, and antioxidants. Other components are poly-unsaturated and saturated fatty acids (linoleic and palmitic acids), present in lower amounts, while minor components such as polyphenols, tocopherols, and pigments (carotenoids and chlorophylls) further enrich its profile [1,2,3,4]. Based on the production process, the olive oils are classified at different grades, reflecting their final quality (e.g., acidity) and unique characteristics. Extra Virgin Olive Oil (EVOO) has superior quality (color, taste, aroma) compared to Virgin Olive Oil (VOO), lampante, refined olive oil, crude olive pomace oil, refined olive pomace oil, and olive pomace oil [5]. EVOO’s high market value, driven by limited availability and demand, makes it vulnerable to adulteration, often involving more affordable edible oils of lower quality [6]. These practices raise ethical and, in some cases, even health problem issues, prompting regulatory agencies [7], organizations [8], and scientists [9,10,11,12] to search and develop practically suitable countermeasures.

Conventional analytical techniques such as Gas Chromatography-Mass Spectrometry (GC-MS) [13,14,15], High-Performance Liquid Chromatography (HPLC) [16,17], and Fourier Transform Infrared Spectroscopy (FTIR) [18,19,20] have been among the main methods used for assessing olive oil quality. GC-MS and HPLC are particularly valued for their ability to identify and quantify the different ingredients present in olive oil, including fatty acids and polyphenols, which are regarded as significant indicators for olive oil characterization. FTIR spectroscopy has also been widely used for compositional analysis due to its ability to identify key chemical bonds and structures. Undoubtedly, these methods have contributed greatly to the assessment of olive oil; nonetheless, they exhibit some limitations. They are typically time-consuming, often requiring extensive sample preparation, while the use of organic solvents renders them environmentally unfriendly. Moreover, they require expensive experimental apparatus and specialized personnel. These limitations often render these methods less accessible for routine applications. Based on these, optical spectroscopic techniques are more practical and efficient alternatives for olive oil analysis.

Recently, Laser-Induced Breakdown Spectroscopy (LIBS) has been demonstrated as being an efficient tool for the rapid and in situ identification of adulteration and the authentication of olive oil samples. LIBS, a laser-based technique combined with emission spectroscopy, can provide the elemental analysis of a sample in real-time, without any prior preparation of the sample [21,22,23,24,25]. This is achieved by the spectral analysis of the light emitted by the micro-plasma that is generated by focusing a strong enough laser beam onto a sample. The interaction of the laser light with the sample results in the ablation and the subsequent atomization of the sample, along with the production of excited atoms/ions and/or small diatomic species (usually), arising from the fragmentation of larger molecular species present in the plasma, which upon de-excitation emit characteristic spectral lines [26,27,28,29]. The latter can reveal the elemental composition of the sample in principle. In that context, Gyftokostas et al. [30], having used LIBS, studied the classification of some different geographical-origin Greek EVOOs; they achieved accuracies of almost 100%. Similarly, Nanou et al. [31] employed LIBS to differentiate some EVOOs from their mixtures with edible oils, achieving prediction accuracies between 91 and 99%. Similarly, UV-Vis-NIR absorption spectroscopy can provide information about the composition of a sample, and the content of the different ingredients it contains. UV-Vis-NIR absorption spectroscopy has also been proposed for olive oil analysis [32,33,34,35,36,37,38]. Although these techniques were applied separately for olive oil analysis and adulteration detection, and the corresponding spectral data were analyzed and processed independently, the latter can be also combined, i.e., in fused form. In that sense, the combined use of LIBS and UV-Vis-NIR absorption spectroscopy can be a more efficient approach, merging the complementary information and strengths of both techniques, with LIBS enabling the recognition/identification of key elements and UV-Vis-NIR absorption spectroscopy providing the content of polyphenols or pigments (carotenoids and chlorophylls), etc. By combining (i.e., fusing) their spectroscopic data, a more complete profile of the sample can be obtained. The resulting dataset can then be effectively used by machine-learning algorithms for the detection of olive oil adulteration and to improve the overall analysis of its quality.

At this point, it is useful to note that machine learning has demonstrated great potential for the analysis of food matrices, which are often intricate due to the presence of diverse natural and/or artificial organic ingredients. Therefore, these matrices frequently exhibit hidden patterns or relationships that are not straightforwardly apparent by traditional analysis methods. For example, in the case of olive oil, machine learning can identify relationships between characteristics, such as type of cultivar, geographical region, and climatic conditions, which all influence olive oil’s composition and characteristics. Furthermore, machine learning can identify subtle spectral differences that may not be observable by the naked eye, which could indicate quality variations or adulteration. The ability to analyze food quality rapidly, non-invasively, and reliably makes machine learning particularly valuable for applications such as routine quality control in foodstuff production.

Due to these advantages, machine learning has been applied in numerous studies for various food quality tasks, including honey adulteration detection [39], wine authentication [40], milk quality assessment [41], and several others. Regarding olive oil analysis, several spectroscopic techniques, including LIBS and UV-Vis-NIR absorption spectroscopy, as well as others such as NIR [42], Fluorescence [43], and Raman spectroscopies [44], have been employed for quality control and adulteration detection issues. However, most of these studies focus on data from individual spectroscopic methods, limiting their ability to capture the full complexity of olive oil samples. The combination of spectral information/data obtained from different spectroscopic techniques remains less explored. The present study, while independently examining and assessing LIBS and UV-Vis-NIR absorption spectroscopy to detect olive oil adulteration, also evaluates the effect of using the combined (fused) LIBS and UV-Vis-NIR absorption spectroscopic data on the detection of olive oil adulteration. To facilitate direct comparisons, the same set of samples was used in all cases. In that view, 40 Greek EVOOs of different geographical origins were blended with some commercial pomace and seed oils, in concentrations from 10 to 90% *w*/*w* (in a total of 144 adulterated samples). The emission and absorption spectra of LIBS and UV-Vis-NIR absorption techniques were analyzed, both separately and combined, by Linear Discriminant Analysis (LDA), Support Vector Machines (SVMs), and Logistic Regression (LR) algorithms, while the generalization and reliability of each algorithm was evaluated using internal and external validation. To the best of our knowledge, this is the first work where LIBS and UV-Vis-NIR absorption spectroscopy techniques are applied and their results concerning the detection of olive oil adulteration are compared, using the same set of oil olive samples.

## 2. Materials and Methods

### 2.1. Samples

For the experiments, 184 samples were used in total. They comprised 40 EVOOs, collected from four different Greek regions well known for their premium quality olive oil, namely Achaia (A), Kalamata (K), Crete (C), and Lesvos (L) (i.e., 10 EVOOs from each one (A1-A10, K1-K10, C1-C10, L1-L10)), and 144 binary mixtures with pomace (P), soybean (SB), corn (C), and sunflower (SF) oils. One EVOO from each of the four geographical regions was used for the preparation of the different concentration mixtures (e.g., AC1-AC9, AP1-AP9, ASB1-ASB9, ASF1-ASF9, and so on). The components of each mixture (i.e., EVOO and edible oil) were weighed, mixed, and stirred thoroughly for 10–15 min for homogenization. Then, 10 mL of each mixture was placed in a dark glass bottle and stored in the refrigerator at a temperature of −2 to −4 °C, while the samples were rested at room temperature for 12 h prior to the measurements. The concentrations of the prepared mixtures were between 10 and 90% *w*/*w*, with steps of 10%. Table 1 presents the samples’ labels and characteristics.

### 2.2. Experimental Setups

For the LIBS measurements, the 1064 nm output from a Q-switched Nd: YAG laser (Quanta-Ray INDI, Spectra Physics, USA) operating at a repetition rate of 1–10 Hz was employed. The pulse duration was 5 ns and the energy per pulse was 80 mJ. The laser beam was directed perpendicularly on the sample’s free surface and was focused with a 150 mm quartz lens. Approximately 1.5–2 mL of oil was placed in a Petri-like dish, while an argon flow was applied on the sample’s surface to reduce oil splashing. A 50 mm lens was used to collect the emitted light, which was then fed to a fiber bundle, connected to a spectrograph. The spectrograph (AvaSpec-ULS4096CL-EVO, Avantes, The Netherlands) had a 10 μm entrance slit, a 300 lines/mm grating, and a CCD detector (4096 pixels). The emission spectra were recorded using time delay (t_d_) and width (t_w_) of 1.28 μs and 1.05 ms, respectively, extending from 200 to 1000 nm. In total, 10 LIBS spectra were obtained for every sample, with each spectrum being the average of 10 measurements.

The absorption spectra of the oil samples, in a 1 mm thick glass cell, were measured with a double-beam UV-VIS-NIR spectrophotometer (Jasco V-670, Jasco, Japan), and they were recorded from 350 to 750 nm, using a step of 0.5 nm. For each sample, 10 spectra were collected (i.e., 10 individual measurements).

### 2.3. Machine Learning

For the analysis of the spectroscopic data, different machine-learning algorithms were tested, including Principal Component Analysis (PCA), Linear Discriminant Analysis (LDA), Support Vector Machines (SVMs), and Logistic Regression (LR) [45,46,47]. The choice of PCA was based on its ability to perform dimensionality reduction, transforming the original variables into fewer orthogonal components, namely the Principal Components (PCs), while retaining most of the dataset’s variance. In addition, PCA is also effective for the identification and extraction of patterns, relationships, similarities, and/or differences between the data. On the other hand, LDA serves both for dimensionality reduction and classification purposes. Therefore, presuming Gaussian distributions for each class, and equal covariance matrices, LDA can determine a projection direction that maximizes the ratio of between-class variance to within-class variance, effectively creating linear decision boundaries for class separation. SVMs are commonly used for classification tasks by finding a hyperplane in an N-dimensional space (where N is the number of features) that maximizes the margin, or distance, between classes. The support vectors, i.e., the data points closest to the hyperplane, play a key role in defining this decision boundary. Lastly, LR is a classification model that estimates class probabilities using a logistic (sigmoid) function to ensure outputs are constrained between 0 and 1.

For each model, hyperparameter tuning was performed to optimize their performance. Therefore, for the PCA, the number of PCs was selected based on prior investigation to retain most of the variance in the data, while reducing dimensionality. In LDA, the key parameters included the solver (e.g., ‘svd’, ‘lsqr’, or ‘eigen’) and the number of components chosen. As for the SVMs, the kernel type (e.g., linear or rbf) was tested, along with tuning of the C and gamma parameters to enhance model performance. Finally, for LR, the penalty parameter (Lasso regularization, L1) was applied, and the solver (e.g., ‘liblinear’ or ‘saga’) was optimized accordingly to improve the model’s efficiency. All algorithmic analyses were implemented in the Python 3.8 programming environment [48], using libraries such as Scikit-Learn, Pandas, and NumPy. It is useful to note that a standard scaler was applied to the data, i.e., subtracting the mean value and then scaling them to unit variance.

To evaluate the robustness and reliability of the models developed, both internal and external validation procedures were applied. For this purpose, the initial dataset was divided into training and test subsets. During the internal validation process, the training subset was used, and a “k-fold” cross-validation method was applied. Specifically, the training data were divided into k subsets, with k-1 used for training and the remaining 1 subset used for testing the model’s performance. This process was repeated k times, yielding a mean classification accuracy and a standard deviation. In this study, k was set to 10. For the external validation, the data from a separate set of samples, not being part of the training process and completely unknown to the algorithms, were used to assess the final trained models. The resulting prediction accuracy was recorded. Additionally, a confusion matrix was constructed for each model, which presented the correct predictions in the diagonal elements and the misclassifications in the off-diagonal elements. Key performance metrics such as precision and recall, derived from the confusion matrix, were also calculated. Precision measures the model’s ability to correctly recognize elements of a class, while recall assesses its ability to accurately distinguish these elements from the rest.

## 3. Results

### 3.1. LIBS and UV-VIS-NIR Absorption Spectra

**Figure 1:**
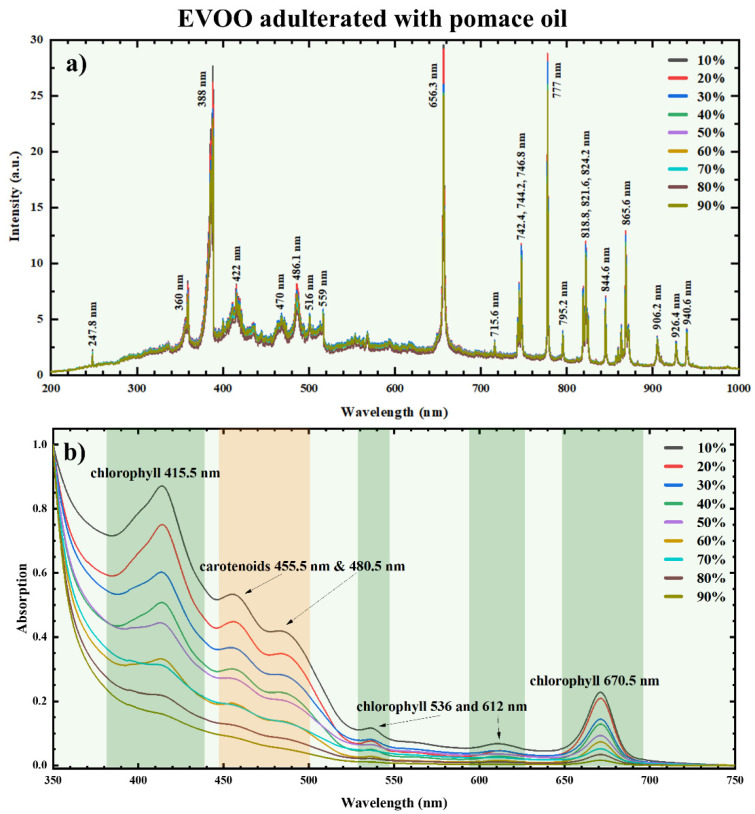
(**a**) LIBS and (**b**) UV-Vis-NIR absorption spectra of an EVOO sample (from the Achaia region) adulterated with pomace oil, in concentrations varying from 10 to 90% *w*/*w*.

The LIBS spectra of an EVOO sample adulterated with pomace oil at different concentrations are illustrated in Figure 1a as an example. As can be seen, several emission lines are observed, with the most prominent ones being the atomic lines of carbon (C), hydrogen (H), oxygen (O), nitrogen (N), and the molecular bands of cyanogen (CN) and C_2_. The assignments of these emission lines were based on the National Institute of Standards and Technology (NIST) spectral database [49] and also on previous studies from our group [30,31]. Table 2 summarizes the most important (for the present work) atomic emission lines and molecular bands observed and the corresponding wavelengths.

The corresponding UV-Vis-NIR absorption spectra are presented in Figure 1b. As can be seen, the characteristic absorption bands of chlorophyll (~415.5, 536, 612, and 670.5 nm) and carotenoids (i.e., 455.5 and 480.5 nm) are clearly observable in these spectra, in full agreement with other studies [50].

It is useful to repeat at this point that the LIBS emission spectra (see, e.g., Figure 1a) and the corresponding UV-Vis-NIR absorption spectra (see, e.g., Figure 1b) provide complementary information about the samples, i.e., their emission and absorption profiles, respectively. However, the former spectra exhibit much smaller variations as the pomace oil concentration increases, while the latter present significant variations, as can be easily observed through a simple inspection of these absorption spectra. In the case of LIBS, this is attributed to the very similar elemental compositions of the EVOOs and the edible oils [31], leading to very small changes in the intensities of the observed atomic emission lines. In contrast, the UV-Vis-NIR absorption spectra, capturing the spectral signatures of the different ingredients present in EVOO, exhibit a clear and progressive reduction of the intensities of the chlorophyll and carotenoids’ characteristic bands increasing pomace oil concentration. Therefore, it becomes clear that the absorption bands corresponding to chlorophyll and carotenoids systematically follow a decreasing trend by increasing the concentration of pomace oil, as the latter contains much less, if any at all, of these ingredients (as can be seen from the absorption spectra shown in Supplementary Figure S1). Therefore, these spectral changes straightforwardly indicate the compositional differences between pure and adulterated EVOOs.

### 3.2. Distinguishing Pure EVOOs from Edible Oils’ Adulterated Ones

At first, the possibility of distinguishing the pure EVOOs from their adulterated counterparts was investigated. All the EVOOs (i.e., A1-A10, K1-K10, C1-C10, L1-L10) were treated as a single class and all their mixtures with edible oils as another class (i.e., AC1-AC9, AP1-AP9, ASB1-ASB9, ASF1-ASF9, KC1-KC9, KP1-KP9, KSB1-KSB9, KSF1-KSF9, CC1-CC9, CP1-CP9, CSB1-CSB9, CSF1-CSF9, LC1-LC9, LP1-LP9, LSB1-LSB9, LSF1-LSF9). The spectral data of LIBS, UV-Vis-NIR absorption, and their fusion were analyzed by the three machine-learning (ML) algorithms. For the analysis and the subsequent validation of the models’ performance, two datasets were built, one for internal validation (i.e., training dataset), comprising 144 samples (i.e., 32 and 112 EVOOs and adulterated ones, respectively), and a second one for external validation (i.e., a test dataset), consisting of 40 samples (i.e., 8 and 32 EVOOs and adulterated ones, respectively). In all cases, the data were pretreated by PCA for dimensionality reduction. Therefore, for the LIBS data, the optimum number of PCs was determined to be between 30 and 70, while for the UV-Vis-NIR absorption data and the fused data, 10–20 and 50–80 PCs, respectively, were found to be sufficient. For each spectroscopic dataset and each algorithm, the determined number of PCs to achieve maximum accuracy is presented in Supplementary Table S1.

The pretreated data were subsequently analyzed by the LDA, SVMs, and LR algorithms. The results of the models using the LIBS, the UV-Vis-NIR absorption data, and the fused data are reported in Table 3. Therefore, the classification accuracies (resulting from the internal validation) and the prediction accuracies (resulting from the external validation) were found to attain remarkably high values for all datasets, ranging between 98–100% and 97–99%, respectively. The obtained results demonstrate the effectiveness of both techniques for discriminating the pure EVOOs from their adulterated counterparts.

A more complete picture concerning the determined prediction accuracies is given by the respective confusion matrices depicted in Table 4, where the rows denote the actual classes and the columns the predicted ones. As can be seen, most of the spectra were successfully classified, and only a few misclassifications were found to occur. In fact, all three models were found to effectively categorize the spectra of the EVOO samples in their respective class, while the few misclassifications observed correspond to the spectra of the adulterated samples (in particular, for the fused data analysis).

An additional evaluation of the successful models’ performance is provided by the precision and the recall scores. The determined values of these metrics for the LDA, SVMs, and LR algorithms, using the LIBS, UV-Vis-NIR absorption, and fused data, are given in Supplementary Table S8. As can be seen in this Table, the values of precision and recall scores were found to range between 0.88 and 1.00 for EVOOS and between 0.98 and 1.00 for the blended samples.

### 3.3. Determination of the Edible Oil Used for Adulteration

**Figure 2:**
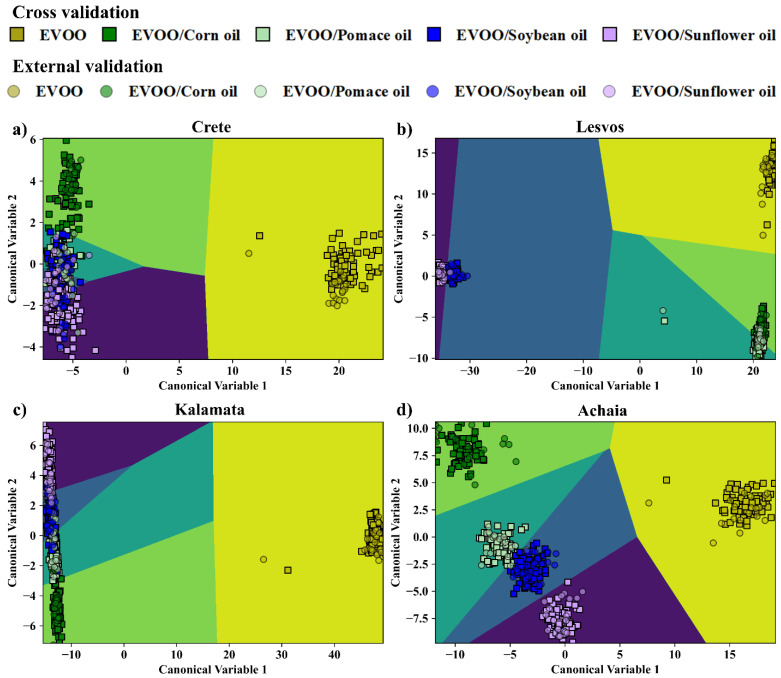
Classification of the EVOO samples and their mixtures with edible oils by LDA, using the LIBS spectral data (emission) for: (**a**) Crete, (**b**) Lesvos, (**c**) Kalamata, and (**d**) Achaia.

**Figure 3:**
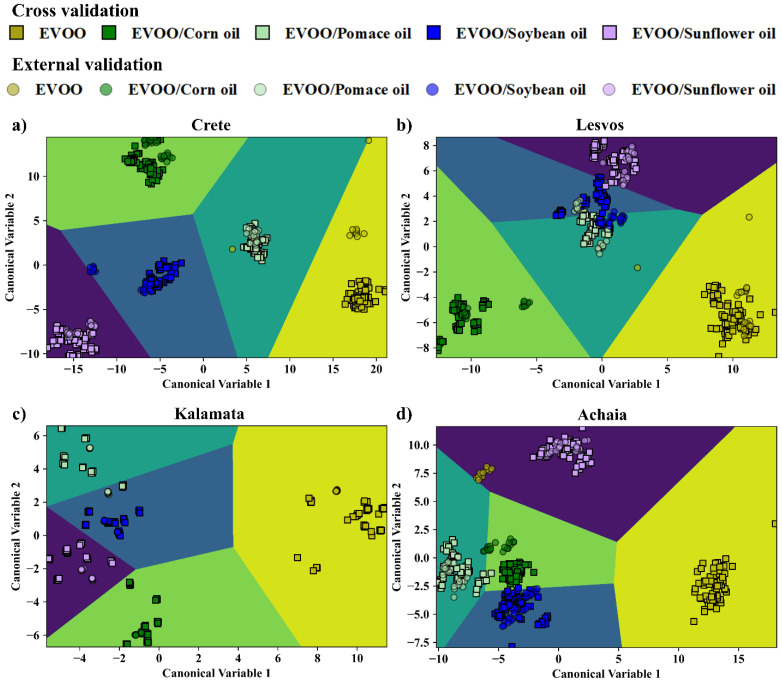
Classification of the EVOO samples and their mixtures with the edible oils by LDA, using the UV-Vis-NIR absorption data for: (**a**) Crete, (**b**) Lesvos, (**c**) Kalamata, and (**d**) Achaia.

**Figure 4:**
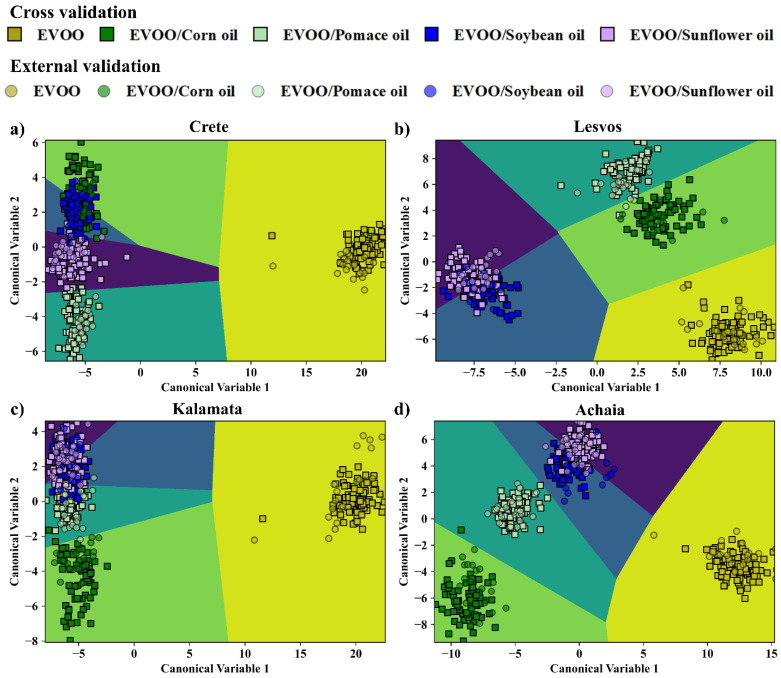
Classification of the EVOO samples and their mixtures with the edible oils by LDA, using the combined/fused data for: (**a**) Crete, (**b**) Lesvos, (**c**) Kalamata, and (**d**) Achaia.

Next, the detection of the edible oil used for EVOOs’ adulteration was attempted. For this purpose, the EVOOs from one geographical area were taken as one class (as, e.g., A1–A10, and so on for the rest), and their adulterated counterparts as four distinct classes (as, e.g., AC1–AC9, AP1–AP9, ASB1–ASB9, ASF1–ASF9), each one corresponding to a single edible oil used for adulteration. Therefore, in total, five classes were formed for each geographical area. Each training dataset contained 36 samples (i.e., 8 EVOOs and 28 adulterated EVOO samples), and each test dataset included 10 samples (i.e., 2 EVOOs and 2 adulterated EVOOs with each edible oil). Again, as in the previous section, the LIBS, UV-Vis-NIR absorption spectroscopy, and the fused data were initially treated by PCA, and the optimum numbers of PCs were determined. In Supplementary Table S1, the complete results of this investigation are presented. The LDA plots of EVOOs’ discrimination from their mixtures with the different edible oils, using the LIBS, UV-Vis-NIR absorption, and combined/fused data, are shown in Figure 2, Figure 3 and Figure 4. The five classes considered are differently colored (i.e., yellow, green, cyan, blue, and purple), while the squares and circles correspond to the training and test data, respectively. The boundaries for discrimination, created by the LDA algorithm, are also plotted and colored accordingly. From the inspection of the LDA plots, all EVOOs are excellently distinguished from the blended samples, in all cases. Also, in general, the mixtures (of the EVOOs with the different edible oils) are well separated between them. Some minor overlapping observed, mainly between the soybean and the sunflower blends, is considered rather insignificant.

The overall results obtained by LDA, using the optimum number of PCs, are presented in Table 3, summarizing the calculated classification and prediction accuracies. As shown, the performance of the LDA for the LIBS data was very successful in all cases, as evidenced by the prediction accuracy values ranging between 94.0 and 99.0%. Slightly better results were obtained from the LDA analysis of the UV-Vis-NIR absorption data, as in this case the prediction accuracy values were found to be between 98.0 and 100%. As for the LDA analysis of the fused data, very satisfactory prediction accuracies were also found, ranging between 93.0 and 100.0%.

The analysis of the spectroscopic data by the SVM algorithms also resulted in exceedingly good results (see also Table 3). In more detail, using the LIBS data, prediction accuracies between 96.0 and 100% were attained while using the UV-Vis-NIR absorption data, and the combined/fused ones of 90–100% and 93.0–97.0% were obtained. Similarly satisfactory results occurred for the LR algorithm, where, by using the LIBS data, prediction accuracies of 94.0–100% were achieved, while the use of the absorption data and the combined data resulted in prediction accuracies of 90.0–100% and 94.0–100%, respectively.

A more detailed insight into the models’ efficiency in discriminating the EVOOs from their adulterated counterparts is provided by the corresponding confusion matrices presented in Table 5, Table 6 and Table 7 (summarizing the classification results for the LDA algorithm) and in Supplementary Tables S2–S7 (summarizing the classification results for the SVMs and LR algorithms). As depicted in these Tables, nearly all the spectral data were correctly assigned to their respective classes. Specifically, the analyses of the LIBS spectra by the different models resulted in very accurate classification, with very few incorrect predictions (i.e., 1 to 5 spectra), mostly for the adulterated samples. Similar observations can be made from the inspection of the confusion matrices of the fused data. In this case, a few misclassifications occurred concerning the soybean oil-mixed EVOO samples, which the models falsely recognized as sunflower oil-mixed EVOO ones. As for the confusion matrices for the absorption spectra, the models correctly predicted most of the EVOO spectra from Crete, Lesvos, and Kalamata, and their corresponding mixtures, while they classified correctly only half of the EVOOs originating from Achaia (i.e., 10 out of 20 spectra).

The precision and recall scores were also determined and are summarized in Supplementary Tables S9–S11. As shown, both metrics attained values of 1, indicating the suitability of the implemented models for recognizing the EVOO samples from their blends, as well as detecting the edible oil used for adulteration.

## 4. Discussion

In this work, LIBS and UV-Vis-NIR absorption spectroscopy, assisted by machine-learning tools, were applied to differentiate some EVOOs from their mixtures with edible oils, and for the identification of the type of edible oil used. The two techniques provide spectral information related to different fundamental properties of the samples, with LIBS providing the elemental composition and UV-Vis-NIR absorption spectroscopy providing its composition (in terms of its molecular constituents/ingredients). The analysis of the collected emission and absorption spectral data by the machine-learning algorithms yielded very similar and highly satisfactory results concerning the discrimination of EVOOs from their mixtures, attaining prediction accuracy as high as 99.8%, as can be seen in Table 3. As for the detection of the edible oil used for the adulteration of the EVOOs, the prediction accuracies obtained by LIBS were found to be between 94.0 and 100.0%, and between 90.0 and 100.0% from the UV-VIS-NIR absorption measurements.

For the analysis of the spectroscopic data (i.e., algorithmic training, etc.), the data of each technique were used separately (i.e., two different datasets) and combined in a single dataset (i.e., fused data) in a low-level data fusion approach. The data fusion approach was applied to investigate its effect on the models’ classification performance, since sometimes data fusion approaches can improve the efficiency of models’ classification accuracy, compared to their performance using the individual datasets. However, in the present case, the analysis using the fused data resulted in very similar prediction accuracies to those obtained by analyzing the LIBS and UV-Vis-NIR absorption spectra separately, yielding accuracy values between 93.0 and 100.0%, while in a few cases even slightly lower accuracies were obtained, evidencing that there is not a general rule that the fused data will always outperform compared to individual datasets. In fact, the effectiveness of fusion analysis can vary based on the context and the conditions under which a study is performed.

Based on the results obtained by the two techniques, it is obvious that they are both very efficient and can operate successfully, attaining exceptional classification accuracies for the discrimination of EVOOs from their mixtures with edible oils and the determination of the edible oil as well. In addition, both techniques are experimentally simple enough, while they do not require any time-consuming sample preparation procedures. However, in general, the LIBS technique operates much faster, since only a few seconds are required to obtain an LIBS spectrum, while the measurement of an absorption spectrum requires few or several minutes (depending on the number of spectra acquired for averaging and the scan speed (nm/s) of the spectrophotometer used). Based on these, it can be supported that the LIBS technique is preferable for on-line and in situ applications concerning olive oil authentication tasks.

Recently, several studies have reported using LIBS, UV-Vis-NIR absorption spectroscopy, Fluorescence spectroscopy, Attenuated Total Reflection Mid-Infrared (ATR-MIR) spectroscopy, and Near Infrared Reflectance spectroscopy (NIR) for EVOO adulteration detection. For example, Caceres et al. [51] utilized LIBS to identify the adulteration of some EVOOs and VOOs with some seed and hazelnut oils. In this study, a Neural Network (NN) algorithm was applied, resulting in discrimination accuracies of up to 95%. As mentioned in the introduction, Nanou et al. [31] have also used LIBS for the analysis of some pure EVOOs and their mixtures with some edible oils, employing LDA, SVMs, LR, and Gradient Boosting (GB) algorithms. Values as high as 99% were reported. In another work, Milanez et al. [52] utilized UV-Vis-NIR absorption spectroscopy to study some EVOOs adulterated with soybean oil, aiming to determine the most suitable spectral features for classification purposes, using a Successive Projections Algorithm (SPA) prior to the implementation of the LDA algorithm. In the same spirit, as an extension of the previous work, Milanez et al. [53] employed UV-Vis-NIR absorption and Fluorescence spectroscopies to detect the adulteration of some EVOO samples with soybean oil. They employed some Partial Least Squares (PLS) and Multiple Linear Regression (MLR) models, which gave R^2^ values of up to 0.98. For similar purposes, Didham et al. [54] employed UV-Vis-NIR absorption and Attenuated Total Reflection Mid-Infrared (ATR-MIR) spectroscopies to analyze some EVOO samples mixed with sunflower and canola oils. They reported R^2^ values of up to 0.98 by implementing PLS Discriminant Analysis (PLS-DA). In another work reported by Castro et al. [55], NIR and UV-Vis-NIR absorption spectroscopies were used to study some olive oil samples blended with refined sunflower, high oleic sunflower, corn, pomace, and seed oils. They reported R^2^ values of up to 0.96 by employing a PLS-DA model.

From the above literature review, it appears that LIBS and UV-Vis-NIR absorption spectroscopy have not been applied and assessed comparatively previously using the same set of samples. Regarding this point, the present work is the first one exploring this issue, to the best of our knowledge, while, in a couple of recent studies employing both these techniques, the focus was on different aspects of olive oil authentication, such as, e.g., the discrimination of EVOOs based on geographical and/or cultivar origin (Gyftokostas et al. [30] and Stefas et al. [35]).

## 5. Conclusions

In this study, the detection of adulteration of some EVOOs with different lower-quality edible oils (i.e., pomace, soybean, corn, and sunflower oils) was investigated by means of LIBS and UV-Vis-NIR absorption spectroscopy. The former technique provides the elemental composition of the sample by recording the emission of a micro-plasma created on its surface, while the latter, measuring the absorption of the different ingredients of the sample (as, e.g., chlorophyll, carotenoids, etc.), reveals its composition. For the measurements, 40 EVOOs originating from four Greek regions (i.e., Crete, Lesvos, Kalamata, and Achaia) renowned for their premium quality EVOOs, and binary mixtures of them with edible oils, at various concentrations (10 to 90% *w*/*w*), were studied. Furthermore, the possibility of identification of the type of edible oil used for adulteration was examined.

For the analyses of the experimental data of each technique, and of the fused data, different machine-learning algorithmic models (e.g., LDA, SVMs, and LR) were employed, while the performance of each model and each technique were thoroughly evaluated and assessed. The obtained results were found to be highly satisfactory, with prediction accuracies attaining values of up to 100%. As an example, using the data obtained by LIBS and UV-Vis-NIR absorption spectroscopy separately, and the fused dataset, the results concerning the discrimination between pure and adulterated EVOOs were found to exceed 99%. As for the determination of the type of adulterant, both techniques and their fused data reached accuracies of up to 100% (as, e.g., in the case of pure Cretan EVOOs (C1-C10) and their adulterated counterparts CC1-CC9, CP1-CP9, CSB1-CSB9, and CSF1-CSF9).

Based on the obtained results (see also Table 3), it becomes evident that both LIBS and UV-Vis-NIR absorption spectroscopy can very efficiently and accurately detect the adulterated EVOOs from the unadulterated ones. From a practical point of view, both LIBS and UV-Vis-NIR absorption spectroscopy are relatively low-cost, simple in operation, and easy to implement, while they can also be portable. Importantly, they do not require any sample preparation. LIBS, in particular, being much faster than UV-Vis-NIR absorption spectroscopy, can be readily used for real-time analysis, allowing for the continuous monitoring of the samples’ quality. All these advantages make LIBS more suitable for routine analysis and quality control in both online and in situ applications.

## Figures and Tables

**Table 1:** Samples’ labels and characteristics.

Sample	Geographical Origin	Adulterant	Adulterant Concentration (% *w*/*w*)
**A**1 to **A**10	**A**chaia	-	-
**K**1 to **K**10	**K**alamata	-	-
**C**1 to **C**10	**C**rete	-	-
**L**1 to **L**10	**L**esvos	-	-
**AC**1 ro **AC**9	**A**chaia	**C**orn	10–90
**AP**1 to **AP**9	**A**chaia	**P**omace	10–90
**ASB**1 to **ASB**9	**A**chaia	**S**oy**b**ean	10–90
**ASF**1 to **ASF**9	**A**chaia	**S**un**f**lower	10–90
**KC**1 to **KC**9	**K**alamata	**C**orn	10–90
**KP**1 to **KP**9	**K**alamata	**P**omace	10–90
**KSB**1 to **KSB**9	**K**alamata	**S**oy**b**ean	10–90
**KSF**1 to **KSF**9	**K**alamata	**S**un**f**lower	10–90
**CC**1 to **CC**9	**C**rete	**C**orn	10–90
**CP**1 to **CP**9	**C**rete	**P**omace	10–90
**CSB**1 to **CSB**9	**C**rete	**S**oy**b**ean	10–90
**CSF**1 to **CSF**9	**C**rete	**S**un**f**lower	10–90
**LC**1 to **LC**9	**L**esvos	**C**orn	10–90
**LP**1 to **LP**9	**L**esvos	**P**omace	10–90
**LSB**1 to **LSB**9	**L**esvos	**S**oy**b**ean	10–90
**LSF**1 to **LSF**9	**L**esvos	**S**un**f**lower	10–90

**Table 2:** Most important atomic emission lines and molecular bands observed in the LIBS spectra of the olive oil samples.

Element	Wavelength (nm)
C (I)	247.8, 795.2, 906.2, 940.6
O (I)	715.6, 777, 844.6, 926.4
N (I)	742.4, 744.2, 746.8, 818.8, 821.6, 824.2, 862.9, 865.6
H_a_ and H_β_	656.3 and 486.1
CN (Δν = +1, 0, −1)	360, 388, and 422
C_2_ (Δν = +1, 0, −1)	470, 516, and 559

**Table 3:** Classification and prediction accuracies obtained by the LDA, SVMs, and LR algorithms, using the LIBS, UV-Vis-NIR absorption, and fused data of EVOOs and their mixtures with edible oils.

		LIBS		UV-Vis-NIR Absorption		Fused Data	
		Classification (%)	Prediction (%)	Classification (%)	Prediction (%)	Classification (%)	Prediction (%)
All EVOOs	LDA	100.0 ± 0.0	99.8	98.3 ± 1.1	97.5	99.8 ± 0.4	98.5
	SVMs	99.9 ± 0.3	99.8	98.3 ± 1.2	99.8	99.8 ± 0.3	99.8
	LR	99.9 ± 0.2	99.8	99.2 ± 0.6	99.8	99.2 ± 0.9	98.8
Crete	LDA	94.7 ± 3.4	94.0	100.0 ± 0.0	98.0	99.7 ± 0.3	100.0
	SVMs	98.6 ± 1.9	100.0	100.0 ± 0.0	100.0	100.0 ± 0.0	94.0
	LR	98.9 ± 1.8	100.0	100.0 ± 0.0	100.0	99.4 ± 1.1	100.0
Lesvos	LDA	97.2 ± 3.0	97.0	96.9 ± 3.0	100.0	97.1 ± 2.2	94.0
	SVMs	95.3 ± 5.1	96.0	99.7 ± 0.9	99.0	99.1 ± 1.3	94.0
	LR	94.7 ± 4.4	97.0	99.7 ± 0.9	100.0	97.1 ± 3.1	94.0
Kalamata	LDA	96.4 ± 2.8	97.0	100.0 ± 0.0	100.0	91.7 ± 3.5	98.0
	SVMs	96.4 ± 3.5	99.0	100.0 ± 0.0	100.0	94.4 ± 3.0	97.0
	LR	93.3 ± 5.4	94.0	99.4 ± 1.1	99.0	92.2 ± 3.7	98.0
Achaia	LDA	98.6 ± 1.9	99.0	99.1 ± 1.3	90.0	93.1 ± 4.5	93.0
	SVMs	98.9 ± 1.8	98.0	99.2 ± 1.3	90.0	93.1 ± 2.6	93.0
	LR	97.2 ± 3.7	98.0	100.0 ± 0.0	90.0	90.8 ± 2.8	97.0

**Table 4:** Confusion matrices constructed for the LDA, SVMs, and LR algorithms, using the LIBS, UV-Vis-NIR absorption, and the fused data, where all EVOOs are treated as one class while all EVOO-edible oil mixtures are treated as another class, (the correct classifications are the diagonal elements (bold characters)).

LDA/SVMs/LR Algorithms			
		Predicted Class	
	Actual Class	EVOOs	Mixtures
**LIBS**	**EVOOs**	**79/79/79**	1/1/1
	**Mixtures**	0/0/0	**320/320/320**
**UV-Vis-NIR absorption**	**EVOOs**	**70/79/79**	10/1/1
	**Mixtures**	0/0/0	**320/320/320**
**Fusion**	**EVOOs**	**74/79/80**	6/1/0
	**Mixtures**	0/0/5	**320/320/315**

**Table 5:** Confusion matrices constructed for the LDA algorithm using the LIBS data, where all the EVOOs from each region are treated as one class, while the EVOO-edible oil mixtures are treated as four classes, (the correct classifications are the diagonal elements (bold characters)).

LDA Algorithm						
Geographical Origin	Actual Class	Predicted Class				
		EVOOs	Corn Oil	Pomace Oil	Soybean Oil	Sunflower Oil
**Crete**	**EVOOs**	**20**	0	0	0	0
	**Corn oil**	0	**20**	0	0	0
	**Pomace oil**	0	0	**18**	0	2
	**Soybean oil**	0	0	0	**20**	0
	**Sunflower oil**	0	1	3	0	**16**
**Lesvos**	**EVOOs**	**20**	0	0	0	0
	**Corn oil**	0	**20**	0	0	0
	**Pomace oil**	0	2	**18**	0	0
	**Soybean oil**	0	0	0	**20**	0
	**Sunflower oil**	0	0	0	1	**19**
**Kalamata**	**EVOOs**	**20**	0	0	0	0
	**Corn oil**	0	**19**	1	0	0
	**Pomace oil**	0	0	**20**	0	0
	**Soybean oil**	0	0	0	**20**	0
	**Sunflower oil**	0	0	0	2	**18**
**Achaia**	**EVOOs**	**20**	0	0	0	0
	**Corn oil**	0	**20**	0	0	0
	**Pomace oil**	0	0	**20**	0	0
	**Soybean oil**	0	0	0	**19**	1
	**Sunflower oil**	0	0	0	0	**20**

**Table 6:** Confusion matrices constructed for the LDA algorithm using the UV-Vis-NIR absorption data, where all the EVOOs from each region are treated as one class, while the EVOO-edible oil mixtures are treated as four classes, (the correct classifications are the diagonal elements (bold characters)).

LDA Algorithm						
Geographical Origin	Actual Class	Predicted Class				
		EVOOs	Corn Oil	Pomace Oil	Soybean Oil	Sunflower Oil
**Crete**	**EVOOs**	**18**	0	2	0	0
	**Corn oil**	0	**20**	0	0	0
	**Pomace oil**	0	0	**20**	0	0
	**Soybean oil**	0	0	0	**20**	0
	**Sunflower oil**	0	0	0	0	**20**
**Lesvos**	**EVOOs**	**20**	0	0	0	0
	**Corn oil**	0	**20**	0	0	0
	**Pomace oil**	0	0	**20**	0	0
	**Soybean oil**	0	0	0	**20**	0
	**Sunflower oil**	0	0	0	0	**20**
**Kalamata**	**EVOOs**	**20**	0	0	0	0
	**Corn oil**	0	**20**	0	0	0
	**Pomace oil**	0	0	**20**	0	0
	**Soybean oil**	0	0	0	**20**	0
	**Sunflower oil**	0	0	0	0	**20**
**Achaia**	**EVOOs**	**10**	0	10	0	0
	**Corn oil**	0	**20**	0	0	0
	**Pomace oil**	0	0	**20**	0	0
	**Soybean oil**	0	0	0	**20**	0
	**Sunflower oil**	0	0	0	0	**20**

**Table 7:** Confusion matrices constructed for the LDA algorithm using the fused data,(where all the EVOOs from each region are treated as one class, while the EVOO-edible oil mixtures are treated as four classes, (the correct classifications are the diagonal elements (bold characters)).

LDA Algorithm						
Geographical Origin	Actual Class	Predicted Class				
		EVOOs	Corn Oil	Pomace Oil	Soybean Oil	Sunflower Oil
**Crete**	**EVOOs**	**20**	0	0	0	0
	**Corn oil**	0	**20**	0	0	0
	**Pomace oil**	0	0	**20**	0	0
	**Soybean oil**	0	0	0	**20**	0
	**Sunflower oil**	0	0	0	0	**20**
**Lesvos**	**EVOOs**	**20**	0	0	0	0
	**Corn oil**	0	**20**	0	0	0
	**Pomace oil**	0	2	**20**	0	0
	**Soybean oil**	0	0	0	**16**	4
	**Sunflower oil**	0	0	0	2	**18**
**Kalamata**	**EVOOs**	**20**	0	0	0	0
	**Corn oil**	0	**20**	0	0	0
	**Pomace oil**	0	0	**20**	0	0
	**Soybean oil**	0	0	0	**19**	1
	**Sunflower oil**	0	0	0	0	**20**
**Achaia**	**EVOOs**	**20**	0	0	0	0
	**Corn oil**	0	**20**	0	0	0
	**Pomace oil**	0	0	**20**	0	0
	**Soybean oil**	0	0	0	**16**	4
	**Sunflower oil**	0	0	0	3	**17**

## Data Availability

The original contributions presented in this study are included in the article/Supplementary Materials. Further inquiries can be directed to the corresponding author.

## Supplementary Materials

The following supporting information can be downloaded at: https://www.mdpi.com/article/10.3390/foods14020321/s1. Figure S1: UV-Vis-NIR absorption spectra of an EVOO sample, pomace, corn, soybean, and sunflower oils; Table S1: Optimum number of PCs used for the analysis of the LIBS, UV-Vis-NIR absorption, and fused data by LDA, SVMs, and LR algorithms; Table S2: Confusion matrices constructed for the SMVs algorithm, using the LIBS data (where all the EVOOs from each region treated as one class while the EVOO-edible oil mixtures treated as four classes); Table S3: Confusion matrices constructed for the LR algorithm, using the LIBS data (where all the EVOOs from each region treated as one class while the EVOO-edible oil mixtures treated as four classes); Table S4: Confusion matrices constructed for the SVMs algorithm, using the UV-Vis-NIR absorption data (where all the EVOOs from each region treated as one class while the EVOO-edible oil mixtures treated as four classes); Table S5: Confusion matrices constructed for the LR algorithm, using the UV-Vis-NIR absorption data (where all the EVOOs from each region treated as one class while the EVOO-edible oil mixtures treated as four classes); Table S6: Confusion matrices constructed for the SVMs algorithm, using the fused data (where all the EVOOs from each region treated as one class while the EVOO-edible oil mixtures treated as four classes); Table S7: Confusion matrices constructed for the LR algorithm, using the fused data (where all the EVOOs from each region treated as one class while the EVOO-edible oil mixtures treated as four classes); Table S8: Precision and recall scores for the LDA, SVMs, and LR algorithms, using the LIBS, UV-Vis-NIR absorption, and fused data (where all EVOOs treated as one class while all EVOO-edible oil mixtures treated as another class); Table S9: Precision and recall scores for the LDA, SVMs, and LR algorithms, using the LIBS data (where all the EVOOs from each region treated as one class while the EVOO-edible oil mixtures treated as four classes); Table S10: Precision and recall scores for the LDA, SVMs, and LR algorithms, using the UV-Vis-NIR absorption data (where all the EVOOs from each region treated as one class while the EVOO-edible oil mixtures treated as four classes); Table S11: Precision and recall scores for the LDA, SVMs, and LR algorithms, using the fused data (where all the EVOOs from each region treated as one class while the EVOO-edible oil mixtures treated as four classes).
