# Supplementary material for: Detection of Adulteration of Extra Virgin Olive Oil via Laser-Induced Breakdown Spectroscopy and Ultraviolet-Visible-Near-Infrared Absorption Spectroscopy: A Comparative Study
Source: Foods. 2025 Jan 18;14(2):321. doi: 10.3390/foods14020321 (PMC11764541; doi:10.3390/foods14020321)
Supplement: Supplementary file 1 [file foods-14-00321-s001.zip › foods-3385666-supplementary.pdf]

Supplementary Material

Detection of Adulteration of Extra Virgin Olive Oil via Laser Induced Breakdown Spectroscopy and UV-Vis-NIR Absorption Spectroscopy: A Comparative Study

Eleni Nanou[1,2], Marios Bekogianni[1], Theodoros Stamatoukos[1], Stelios Couris[1,2,*]

[1] University of Patras, Department of Physics, Patras, 26504, Greece

[2] Institute of Chemical Engineering Sciences (ICE-HT), Foundation for Research and Technology-Hellas (FORTH), Patras, 26504, Greece

*couris@upatras.gr

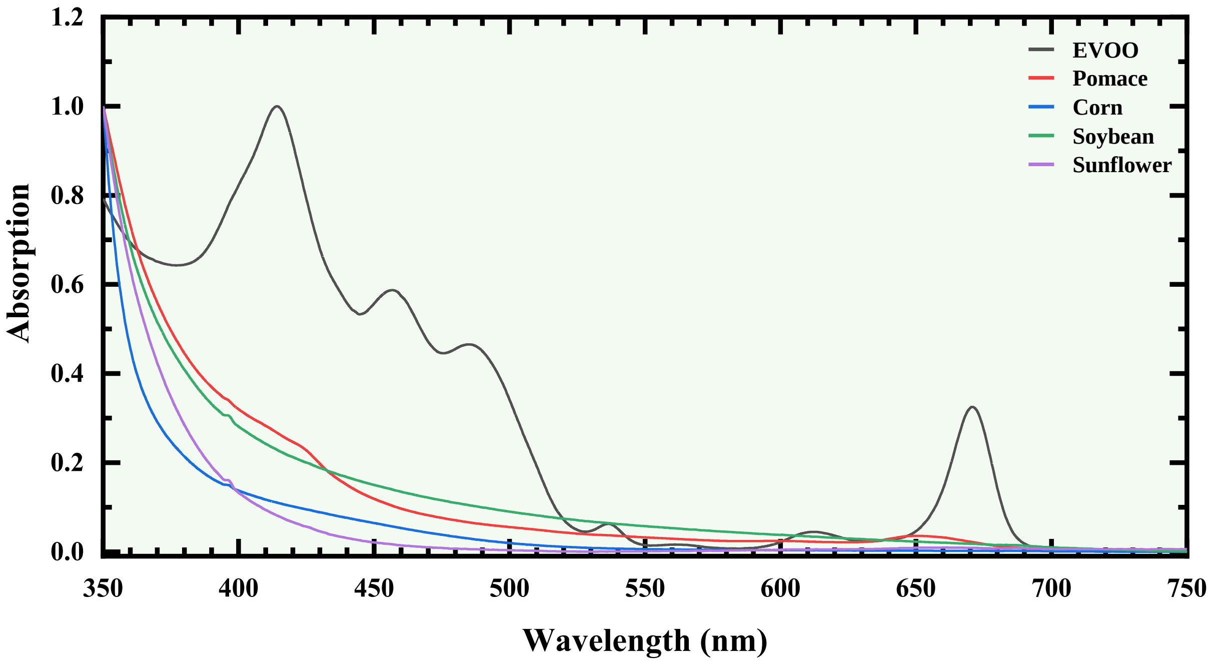

Figure S1. UV-Vis-NIR absorption spectra of an EVOO sample, pomace, corn, soybean, and sunflower oils

Table S1. Optimum number of PCs used for the analysis of the LIBS, UV-Vis-NIR absorption, and fused data by LDA, SVMs, and LR algorithms

		Optimum No. of PCs		
		**LIBS**	**UV-Vis-NIR absorption**	**Fused data**
**All EVOOs and mixtures**	**LDA**	70	20	60
	**SVMs**	30	10	50
	**LR**	30	10	80

EVOOs from Crete and mixtures	LDA	20	20	20
	SVMs	40	10	40
	LR	40	10	20
EVOOs from Lesvos and mixtures	LDA	40	10	20
	SVMs	20	20	30
	LR	20	10	60
EVOOs from Kalamata and mixtures	LDA	70	10	40
	SVMs	80	10	30
	LR	40	20	20
EVOOs from Achaia and mixtures	LDA	30	20	90
	SVMs	70	20	70
	LR	110	10	100

Table S2. Confusion matrices constructed for the SMVs algorithm, using the LIBS data (where all the EVOOs from each region treated as one class while the EVOO-edible oil mixtures treated as four classes)

SVMs algorithm						
**Geographical origin**	**Actual class**	**Predicted class**				
		**EVOOs**	**Corn oil**	**Pomace oil**	**Soybean oil**	**Sunflower oil**
**Crete**	**EVOOs**	**20**	0	0	0	0
	**Corn oil**	0	**20**	0	0	0
	**Pomace oil**	0	0	**20**	0	0
	**Soybean oil**	0	0	0	**20**	0
	**Sunflower oil**	0	0	0	0	**20**
**Lesvos**	**EVOOs**	**19**	1	0	0	0
	**Corn oil**	0	**18**	2	0	0
	**Pomace oil**	0	0	**20**	0	0
	**Soybean oil**	0	0	0	**20**	0
	**Sunflower oil**	0	0	0	1	**19**
**Kalamata**	**EVOOs**	**20**	0	0	0	0
	**Corn oil**	0	**20**	0	0	0
	**Pomace oil**	0	0	**20**	0	0
	**Soybean oil**	0	0	0	**20**	0
	**Sunflower oil**	0	0	0	1	**19**

**Achaia**	**EVOOs**	**20**	0	0	0	0
	**Corn oil**	0	**20**	0	0	0
	**Pomace oil**	0	0	**19**	1	0
	**Soybean oil**	0	0	1	**19**	0
	**Sunflower oil**	0	0	0	0	**20**

Table S3. Confusion matrices constructed for the LR algorithm, using the LIBS data (where all the EVOOs from each region treated as one class while the EVOO-edible oil mixtures treated as four classes)

**LR algorithm**						
**Geographical origin**	**Actual class**	**Predicted class**				
		**EVOOs**	**Corn oil**	**Pomace oil**	**Soybean oil**	**Sunflower oil**
**Crete**	**EVOOs**	**20**	0	0	0	0
	**Corn oil**	0	**20**	0	0	0
	**Pomace oil**	0	0	**20**	0	0
	**Soybean oil**	0	0	0	**20**	0
	**Sunflower oil**	0	3	0	0	**20**
**Lesvos**	**EVOOs**	**19**	1	0	0	0
	**Corn oil**	0	**19**	0	1	0
	**Pomace oil**	0	0	**20**	0	0
	**Soybean oil**	0	0	0	**20**	0
	**Sunflower oil**	0	0	0	1	**19**
**Kalamata**	**EVOOs**	**20**	0	0	0	0
	**Corn oil**	0	**18**	2	0	0
	**Pomace oil**	0	0	**20**	0	0
	**Soybean oil**	0	0	0	**19**	1
	**Sunflower oil**	0	0	0	3	**17**
**Achaia**	**EVOOs**	**20**	0	0	0	0
	**Corn oil**	0	**20**	0	0	0
	**Pomace oil**	0	0	**20**	0	0
	**Soybean oil**	0	0	1	**18**	1
	**Sunflower oil**	0	0	0	0	**20**

Table S4. Confusion matrices constructed for the SVMs algorithm, using the UV-Vis-NIR absorption data (where all the EVOOs from each region treated as one class while the EVOO-edible oil mixtures treated as four classes)

SVMs algorithm						
**Geographical origin**	**Actual class**	**Predicted class**				
		**EVOOs**	**Corn oil**	**Pomace oil**	**Soybean oil**	**Sunflower oil**
**Crete**	**EVOOs**	**20**	0	0	0	0
	**Corn oil**	0	**20**	0	0	0
	**Pomace oil**	0	0	**20**	0	0
	**Soybean oil**	0	0	0	**20**	0
	**Sunflower oil**	0	0	0	0	**20**
**Lesvos**	**EVOOs**	**19**	0	0	1	0
	**Corn oil**	0	**20**	0	0	0
	**Pomace oil**	0	0	**20**	0	0
	**Soybean oil**	0	0	0	**20**	0
	**Sunflower oil**	0	0	0	0	**20**
**Kalamata**	**EVOOs**	**20**	0	0	0	0
	**Corn oil**	0	**20**	0	0	0
	**Pomace oil**	0	0	**20**	0	0
	**Soybean oil**	0	0	0	**20**	0
	**Sunflower oil**	0	0	0	0	**20**
**Achaia**	**EVOOs**	**10**	0	10	0	0
	**Corn oil**	0	**20**	0	0	0
	**Pomace oil**	0	0	**20**	0	0
	**Soybean oil**	0	0	0	**20**	0
	**Sunflower oil**	0	0	0	0	**20**

Table S5. Confusion matrices constructed for the LR algorithm, using the UV-Vis-NIR absorption data (where all the EVOOs from each region treated as one class while the EVOO-edible oil mixtures treated as four classes)

LR algorithm						
**Geographical origin**	**Actual class**	**Predicted class**				
		**EVOOs**	**Corn oil**	**Pomace oil**	**Soybean oil**	**Sunflower oil**
**Crete**	**EVOOs**	**20**	0	0	0	0
	**Corn oil**	0	**20**	0	0	0

	**Pomace oil**	0	0	**20**	0	0
	**Soybean oil**	0	0	0	**20**	0
	**Sunflower oil**	0	0	0	0	**20**
**Lesvos**	**EVOOs**	**19**	0	0	1	0
	**Corn oil**	0	**20**	0	0	0
	**Pomace oil**	0	0	**20**	0	0
	**Soybean oil**	0	0	0	**20**	0
	**Sunflower oil**	0	0	0	0	**20**
**Kalamata**	**EVOOs**	**19**	0	0	1	0
	**Corn oil**	0	**20**	0	0	0
	**Pomace oil**	0	0	**20**	0	0
	**Soybean oil**	0	0	0	**20**	0
	**Sunflower oil**	0	0	0	0	**20**
**Achaia**	**EVOOs**	**10**	0	10	0	0
	**Corn oil**	0	**20**	0	0	0
	**Pomace oil**	0	0	**20**	0	0
	**Soybean oil**	0	0	0	**20**	0
	**Sunflower oil**	0	0	0	0	**20**

Table S6. Confusion matrices constructed for the SVMs algorithm, using the fused data (where all the EVOOs from each region treated as one class while the EVOO-edible oil mixtures treated as four classes)

**SVMs algorithm**						
**Geographical origin**	**Actual class**	**Predicted class**				
		**EVOOs**	**Corn oil**	**Pomace oil**	**Soybean oil**	**Sunflower oil**
**Crete**	**EVOOs**	**20**	0	0	0	0
	**Corn oil**	0	**20**	0	0	0
	**Pomace oil**	0	0	**20**	0	0
	**Soybean oil**	0	0	0	**14**	6
	**Sunflower oil**	0	0	0	0	**20**
**Lesvos**	**EVOOs**	**20**	0	0	0	0
	**Corn oil**	1	**19**	0	0	0
	**Pomace oil**	0	0	**20**	0	0

	**Soybean oil**	0	2	0	**15**	3
	**Sunflower oil**	0	0	0	0	**20**
**Kalamata**	**EVOOs**	**20**	0	0	0	0
	**Corn oil**	0	**20**	0	0	0
	**Pomace oil**	0	0	**20**	0	0
	**Soybean oil**	0	0	0	**17**	3
	**Sunflower oil**	0	0	0	0	**20**
**Achaia**	**EVOOs**	**20**	0	0	0	0
	**Corn oil**	0	**20**	0	0	0
	**Pomace oil**	0	0	**16**	0	0
	**Soybean oil**	0	0	0	**19**	1
	**Sunflower oil**	0	0	0	2	**18**

Table S7. Confusion matrices constructed for the LR algorithm, using the fused data (where all the EVOOs from each region treated as one class while the EVOO-edible oil mixtures treated as four classes)

**LR algorithm**						
**Geographical origin**	**Actual class**	**Predicted class**				
		**EVOOs**	**Corn oil**	**Pomace oil**	**Soybean oil**	**Sunflower oil**
**Crete**	**EVOOs**	**20**	0	0	0	0
	**Corn oil**	0	**20**	0	0	0
	**Pomace oil**	0	0	**20**	0	0
	**Soybean oil**	0	0	0	**20**	0
	**Sunflower oil**	0	0	0	0	**20**
**Lesvos**	**EVOOs**	**20**	0	0	0	0
	**Corn oil**	0	**18**	2	0	0
	**Pomace oil**	0	0	**20**	0	0
	**Soybean oil**	0	0	0	**17**	3
	**Sunflower oil**	0	0	0	1	**19**
**Kalamata**	**EVOOs**	**20**	0	0	0	0
	**Corn oil**	0	**20**	0	0	0
	**Pomace oil**	0	0	**20**	0	0
	**Soybean oil**	0	0	0	**20**	0

	Sunflower oil	0	0	0	2	**18**
Achaia	EVOOs	**20**	0	0	0	0
	Corn oil	0	**20**	0	0	0
	Pomace oil	0	0	**20**	0	0
	Soybean oil	0	0	0	**18**	2
	Sunflower oil	0	0	0	1	**19**

Table S8. Precision and recall scores for the LDA, SVMs, and LR algorithms, using the LIBS, UV-Vis-NIR absorption, and fused data (where all EVOOs treated as one class while all EVOO-edible oil mixtures treated as another class)

			LIBS		UV-Vis-NIR absorption		Fusion	
			Precision	Recall (Sensitivity)	Precision	Recall (Sensitivity)	Precision	Recall (Sensitivity)
All EVOOs and mixtures	LDA	EVOOs	1	0.99	1	0.88	1	0.93
		Mixtures	1	1	0.97	1	0.98	1
	SVMs	EVOOs	1	0.99	1	0.99	1	0.99
		Mixtures	1	1	1	1	1	1
	LR	EVOOs	1	0.99	1	0.99	0.94	1
		Mixtures	1	1	1	1	1	0.98

Table S9. Precision and recall scores for the LDA, SVMs, and LR algorithms, using the LIBS data (where all the EVOOs from each region treated as one class while the EVOO-edible oil mixtures treated as four classes)

			LDA		SVMs		LR	
			Precision	Recall (Sensitivity)	Precision	Recall (Sensitivity)	Precision	Recall (Sensitivity)
LIBS	Crete	EVOOs	1	1	1	1	1	1
		Corn oil	1	1	1	1	1	1
		Pomace oil	0.9	0.9	1	1	1	1
		Soybean oil	1	1	1	1	1	1
		Sunflower oil	0.9	0.8	1	1	1	1
	Lesvos	EVOOs	1	1	1	0.95	1	0.95
		Corn oil	0.9	1	0.95	0.9	0.95	0.95
		Pomace oil	1	0.9	0.91	1	1	1
		Soybean oil	1	1	0.95	1	0.91	1

	Sunflower oil	1	0.9	1	0.95	1	0.95
Kalamata	EVOOs	1	1	1	1	1	1
	Corn oil	1	0.9	1	1	1	0.9
	Pomace oil	1	1	1	1	0.91	1
	Soybean oil	0.9	1	0.95	1	0.86	0.95
	Sunflower oil	1	0.9	1	0.95	0.94	0.85
Achaia	EVOOs	1	1	1	1	1	1
	Corn oil	1	1	1	1	1	1
	Pomace oil	1	1	0.95	0.95	0.95	1
	Soybean oil	1	0.9	0.95	0.95	1	0.9
	Sunflower oil	1	1	1	1	0.95	1

Table S10. Precision and recall scores for the LDA, SVMs, and LR algorithms, using the UV-Vis-NIR absorption data (where all the EVOOs from each region treated as one class while the EVOO-edible oil mixtures treated as four classes)

			LDA		SVMs		LR	
			Precision	Recall (Sensitivity)	Precision	Recall (Sensitivity)	Precision	Recall (Sensitivity)
UV-Vis-NIR absorption	Crete	EVOOs	1	0.9	1	1	1	1
		Corn oil	1	1	1	1	1	1
		Pomace oil	0.9	1	1	1	1	1
		Soybean oil	1	1	1	1	1	1
		Sunflower oil	1	1	1	1	1	1
	Lesvos	EVOOs	1	1	1	0.95	1	1
		Corn oil	1	1	1	1	1	1
		Pomace oil	1	1	1	1	1	1
		Soybean oil	1	1	0.95	1	1	1
		Sunflower oil	1	1	1	1	1	1
	Kalamata	EVOOs	1	1	1	1	1	0.95
		Corn oil	1	1	1	1	1	1
		Pomace oil	1	1	1	1	1	1
		Soybean oil	1	1	1	1	0.95	1
		Sunflower oil	1	1	1	1	1	1
	Achaia	EVOOs	1	0.5	1	0.5	1	0.5
		Corn oil	1	1	1	1	1	1
		Pomace oil	0.7	1	0.67	1	1	1
		Soybean oil	1	1	1	1	0.67	1
		Sunflower oil	1	1	1	1	1	1

Table S11. Precision and recall scores for the LDA, SVMs, and LR algorithms, using the fused data (where all the EVOOs from each region treated as one class while the EVOO-edible oil mixtures treated as four classes)

			LDA		SVMs		LR	
			Precision	Recall (Sensitivity)	Precision	Recall (Sensitivity)	Precision	Recall (Sensitivity)
Fusion	Crete	EVOOs	1	1	1	1	1	1
		Corn oil	1	1	1	1	1	1
		Pomace oil	1	1	1	1	1	1
		Soybean oil	1	1	1	0.7	1	1
		Sunflower oil	1	1	0.77	1	1	1
	Lesvos	EVOOs	1	1	0.95	1	1	1
		Corn oil	1	1	0.90	0.95	1	0.9
		Pomace oil	1	1	1	1	0.91	1
		Soybean oil	0.9	0.8	1	0.75	0.94	0.85
		Sunflower oil	0.8	0.9	0.87	1	0.86	0.95
	Kalamata	EVOOs	1	1	1	1	1	1
		Corn oil	1	1	1	1	1	1
		Pomace oil	1	1	1	1	1	1
		Soybean oil	1	0.9	1	0.85	0.91	1
		Sunflower oil	1	1	0.87	1	1	0.9
	Achaia	EVOOs	1	1	1	1	1	1
		Corn oil	1	1	0.83	1	1	1
		Pomace oil	1	1	1	0.8	1	1
		Soybean oil	0.8	0.8	0.90	0.95	0.95	0.9
		Sunflower oil	0.8	0.8	0.95	0.9	0.90	0.95
